# Effect of Heat–Moisture Treatment on the Structure and Digestibility of Sweet Potato Starch

**DOI:** 10.3390/foods12163076

**Published:** 2023-08-16

**Authors:** Yangyang Sun, Renbing Qin, Jie Zeng, Guanglei Li

**Affiliations:** School of Food Science, Henan Institute of Science and Technology, Xinxiang 453003, China; sy574240030@163.com (Y.S.); qinrenbing@163.com (R.Q.); zengjie623@163.com (J.Z.)

**Keywords:** sweet potato starch, heat–moisture treatment, structure, digestibility

## Abstract

The objective of this study was to investigate the effect of temperature changes during heat–moisture treatment (HMT) on the appearance, structure and digestibility of sweet potato starch (SPS). The results showed that after HMT, there were depressions, cavities and fragments on the surface of SPS particles. The polarized crosses of SPS were irregular and partially blurred. The relative crystallinity and short-range order of SPS decreased, while rearrangement and reorientation of the starch molecules occurred and the thermal stability increased. The resistant starch content of SPS reached the highest (24.77%) after 4 h treatment at 110 °C and 25% moisture. The obtained results can provide a reference for the modification of SPS.

## 1. Introduction

People’s poor eating habits have led to the incidence rates of chronic diseases rising year on year, tending to affect younger individuals [1,2]. The use of specific methods to process raw food materials to change their structure and functional properties can play a role in the prevention and treatment of chronic diseases, which has become a hot spot in modern food research [3].

Sweet potato (*Ipomoea batatas*) is an annual or perennial herb of Convolvulaceae [4]. It is an important medicine and food crop and is the sixth largest crop in the world [5,6]. Due to the advantages of strong disaster resistance, wide adaptability, excellent stability and high yield, sweet potato has been widely planted in China, which has the largest planting area of sweet potato [7,8]. Sweet potatoes are mainly used for the production of food, feed, ethanol and starch [9]. However, among the many types of starch, sweet potato starch (SPS) is a high glycemic index (GI) starch, which can lead to high postprandial blood glucose levels and induce chronic diseases such as hypertension, diabetes and obesity [2,10].

Heat–moisture treatment (HMT) technology was first proposed by Sair in 1967 [11]. It has the advantages of safety and simplicity and does not produce pollutants or require exogenous chemicals. The modification of starch by HMT mainly utilizes the synergistic effect of water and heat. Starch is treated for a specific time (15 min~16 h) at a low moisture content (10~35%) and high temperature (90~130 °C), promoting the rearrangement of molecular chains inside starch particles, changing the structure and physicochemical properties of starch and reducing the content of rapidly digestible starch (RDS) [12,13].

At present, studies on HMT of starch are mainly focused on rice starch and wheat starch [14,15]. There are also many reports on the physicochemical properties and digestibility of SPS. Huang et al. modified SPS with HMT and found that the content of slowly digestible starch (SDS) in SPS increased with the increase in treatment time [16]. Liu et al. studied the effect of moisture change on the gel properties and structure of SPS during HMT; they found that after HMT, the hardness, elasticity and chewability of vermicelli made of SPS were enhanced and the quality of the vermicelli was superior to that of vermicelli made of raw starch [17]. Zhang et al. found that the change in the properties of sweet potato flour was mainly caused by its structural reorganization [10]. In contrast to SPS, sweet potato flour contains many other nutrients. In view of this, the effect of temperature changes on the structure and digestibility of SPS during HMT were evaluated in this study. The obtained results can provide a theoretical basis to further promote the application of SPS.

## 2. Materials and Methods

### 2.1. Chemicals

SPS was purchased from Haihua Shunda Grain and Oil Seasoning Co., Ltd. (Cangzhou, China). Its protein, fat, moisture, mineral and starch contents were 0.22 ± 0.03%, 0.16 ± 0.01%, 12.77 ± 0.26%, 0.11 ± 0.01% and 84.52 ± 0.17%, respectively. Its amylose content was 27.73 ± 0.85%. α-Amylase from porcine pancreas (50 U/mg) and glucoamylase (80 U/mg) were obtained from Bomei Biotechnology Co., Ltd. (Hefei, China). Other chemicals (anhydrous ethanol, sodium hydroxide, glacial acetic acid, anhydrous sodium acetate, etc.) were of analytical grade.

### 2.2. Heat–Moisture Treatment (HMT)

According to the method of Zhang et al. [10] with slight modifications, SPS was mixed with water and sealed in a container at 4 °C for 24 h until its moisture content was adjusted to 25%. The obtained SPS was incubated at 100 °C, 110 °C, 120 °C and 130 °C for 4 h, respectively. The cooled sample was kept at 45 °C for 24 h using a DHA-9140 constant temperature blast drying oven (Shanghai Sanfa Scientific Instrument Co., Ltd., Shanghai, China) and then crushed through a 100 mesh sieve by a 304 high-speed multi-function pulverizer (Beijing Puxi General Instrument Co., Ltd., Beijing, China). The resulting samples were named HMT-100, HMT-110, HMT-120 and HMT-130, respectively.

### 2.3. Observation of Starch Morphology

#### 2.3.1. Scanning Electron Microscopy (SEM) Observations

According to the method of Jhan et al. [18] with slight modifications, a small amount of SPS sample was glued to conductive double-sided adhesive on a loading platform. In the vacuum state, the samples were sprayed with gold by an ion sputtering instrument. An FEI Quanta 200 environmental scanning electron microscope (Hillsboro, OH, USA) was used to record representative images of starch granules at a magnification of 2000 times at a voltage of 15 V.

#### 2.3.2. Polarization Microscope Observation

A 0.65% starch solution was dripped on the slide, covered with glass, and placed on the carrier platform. The polarization properties of starch were observed by a BH200P polarizing microscope (Shanghai Shunyu Hengping Scientific Instrument Co., Ltd., Shanghai, China) under polarized light amplification (200 times).

### 2.4. Determination of Crystal Structure

An appropriate amount of the SPS sample with pre-balanced moisture was tiled in the sample pool, and the excess sample was scraped to ensure a crack-free sample surface and placed on the XRD sample table. The crystal structure of starch was determined by a D8 Advance X-ray diffractometer, (Bruker, Germany). The XRD pattern of the sample in the 2θ range of 4~40° was recorded with a scanning rate of 2°/min at 40 kV and 40 mA. The crystallinity of the samples was calculated by MDI jade 6.0 software [19].

### 2.5. Evaluation of Short-Range Ordered Structures

The short-range ordered structure of the sample was evaluated based on the method of Wang et al. [20]; 1 mg of sample and 100 mg of KBr were mixed and ground in an agate mortar and pressed into a thin disc. The disc was placed in a TENSOR 27 infrared spectrometer (Bruker, Germany). The IR spectrum in the range of 4000–400 cm^−1^ was obtained after scanning 32 times with KBr as the background. The IR spectrum was convoluted by OMNIC software (v.9.2; Thermo Scientific, Madison, WI, USA). The spectrum (1200–800 cm^−1^) was selected with an enhancement factor of 1.9 and a half-peak width of 19 cm^−1^. Peakfit 4.12 was used for peak separation. Then, the intensity ratio of the peaks at 1047 cm^−1^ and 1045 cm^−1^ was calculated.

### 2.6. Measurement of Thermal Properties

The thermal parameters of the sample were measured by a Q2000 differential scanning calorimeter (TA Corporation, New Castle County, Delaware, America) [21]. The sample (5 mg) and deionized water (15 μL) were placed in a crucible, sealed and kept at 4 °C for 24 h. During the determination, the sealed blank crucible was used as the background, nitrogen was used as the carrier gas and the sample was heated at 10 °C/min. The test temperature range was 30 °C~130 °C. The melting start temperature (T_0_), peak temperature (T_p_) and end temperature (T_c_) of the test samples were recorded.

### 2.7. Determination of In Vitro Digestibility of SPS

The in vitro digestibility of the SPS sample before and after HMT was determined according to the method of Englyst [22]. The dried starch sample (0.2 g) was placed in a 50 mL centrifuge tube and mixed with 15 mL of 0.1 mol/L sodium acetate buffer solution (pH = 5.2). The mixture was heated (>90 °C) and gelatinized for 10 min. When the starch solution was cooled to 37 °C, a 10 mL mixed solution of α-amylase from porcine pancreas (290 U/mL) and glucoamylase (15 U/mg) were added. The mixture was kept in a water bath oscillator to simulate the in vitro digestion process (37 °C, 50 r/min). At 0 min, 20 min and 120 min, 0.5 mL of the above solution was mixed with 4 mL of ethanol and centrifuged at 6000 r/min for 10 min. The supernatant (1 mL) was mixed with 1 mL of DNS solution and heated in a boiling water bath for 2 min. The absorbance at 540 nm was read. The absorbance of the mixture of 1 mL distilled water and 1 mL DNS solution at 540 nm was also measured as a blank. The equations for the contents of rapidly digestible starch (RDS), slowly digestible starch (SDS) and resistant starch (RS) in SPS are as follows:

$$RDS(\%)=\frac{(G_{20}-FG)\times 0.9}{TS}\times 100$$

$$SDS(\%)=\frac{(G_{120}-G_{20})\times 0.9}{TS}\times 100$$

RS(%)=(1−RDS−SDS)×100

where *G*_20_ and *G*_120_ are the glucose contents after enzymatic hydrolysis of 20 min and 120 min, respectively; FG is the glucose content in the sample before enzymatic hydrolysis; and TS is the total starch content in the sample.

### 2.8. Statistical Analysis

All tests were repeated 3 times and results were expressed as means ± standard deviation (SD). SPSS 26.0 software was used for single factor variance statistical analysis, and Origin 2017 software was used for plotting. The statistical comparison was based on the Tukey method with a confidence level of 95% (*p* < 0.05).

## 3. Results and Discussion

### 3.1. Effect of HMT on the Morphology of SPS

**Figure 1:**
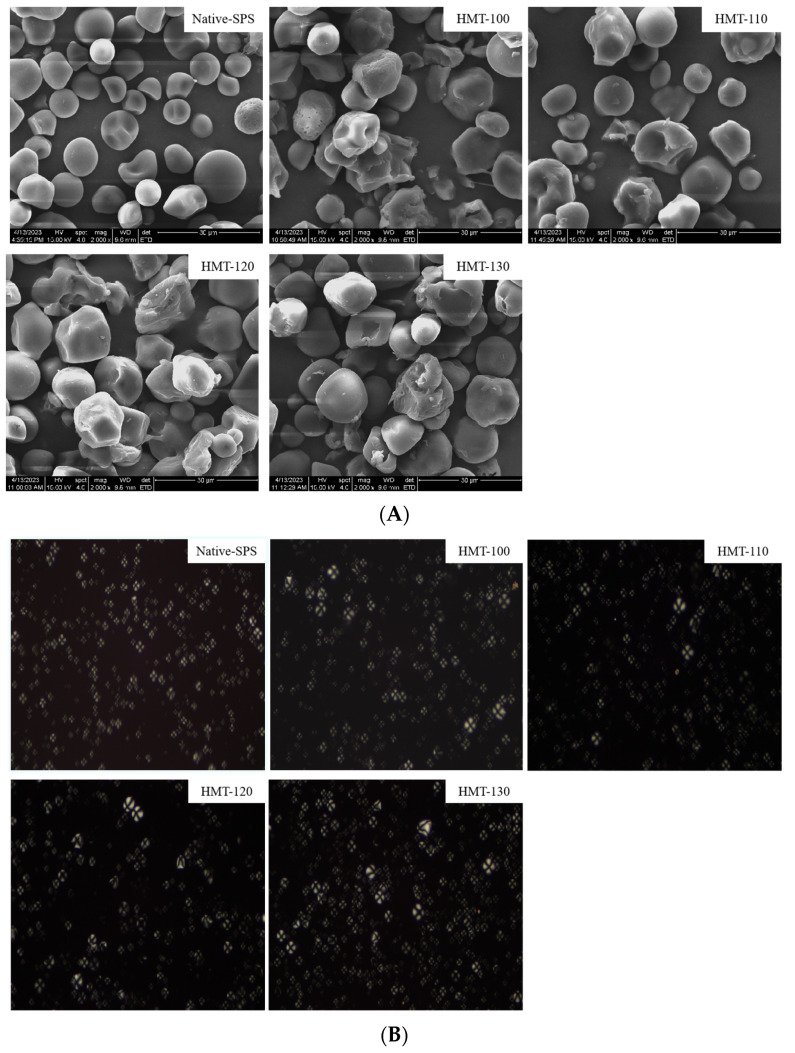
SEM (**A**) and polarizing microscope images (**B**) of SPS before and after HMT. (**A**) SEM images of SPS before and after HMT (2000×); (**B**) polarization microscope images of SPS before and after HMT (200×).

Figure 1 shows the scanning electron microscope and polarizing microscope images of SPS before and after HMT. For starch, its internal structure includes two major regions: crystal and amorphous. The granule structure information of SPS could be obtained by scanning electron microscopy and polarized cross observation. As shown in Figure 1A, it was observed that the granules of raw SPS were intact, with a smooth surface, and were circular or elliptical in shape. After HMT, the surface of SPS particles showed varying degrees of depression and aggregation between particles. As the processing temperature increased, the degree of indentation and destruction of the sample increased, while the aggregation degree of particles first increased (<120 °C) and then decreased (130 °C) due to the rapid dehydration of SPS particles in high-temperature environments and the destruction of stable structures [23].

The polarization cross is a reflection of the birefringence pattern of starch, which can be observed through a polarizing microscope. As shown in Figure 1B, after HMT, the polarization cross of SPS changed obviously, and as the temperature increased (120 °C), its polarization cross became less obvious. This may be due to the high moisture and heat temperature acting on the surface of SPS particles during the HMT process, resulting in the destruction of the amorphous region structure [24].

From this, it could be concluded that the effect of HMT on the morphology of SPS particles was positively correlated with the HMT temperature. With the increase in temperature, the damaging effect on the surface of SPS was greater. When the temperature of HMT was higher than 110 °C, it had a significant destructive effect on the appearance of starch particles, resulting in the depression of the surface of starch particles and a change in the polarization cross.

### 3.2. Effect of HMT on the Crystal Structure of SPS

Starch particles are a semi crystalline system composed of crystalline and amorphous regions; their crystal structure can be characterized by X-ray diffraction patterns. The sharp diffraction peaks in the X-ray curve can reflect the crystal type, crystal size and long-range order of the crystal region. The amorphous region of starch can be reflected by dispersion diffraction on the X-ray curve [25].

**Figure 2:**
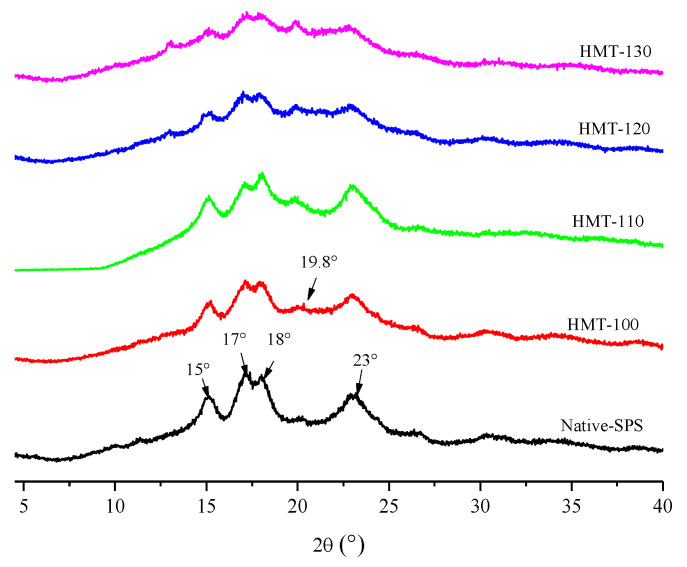
X-ray diffraction patterns of SPS before and after HMT.

The X-ray diffraction patterns of SPS before and after HMT are exhibited in Figure 2. 

Native SPS had strong diffraction peaks at 15°, 17°, 18° and 23°, which belong to A-type starch. After HMT, the characteristic diffraction peaks of SPS were still retained, indicating that HMT did not change the crystal type of SPS, but the diffraction peak at 19.8° changed in varying degrees, which was consistent with the results of Chen et al. [26].

Table 1 demonstrates the changes in the relative crystallinity of SPS before and after HMT. With the increasing temperature, the relative crystallinity of SPS gradually declined. The transfer of heat and water during HMT promoted the breaking of hydrogen bonds between molecular chains in starch granules, changed the orientation, ordered the arrangement, compacted the double helix of starch, and even broke the double helix structure, resulting in the destruction of the crystal structure of SPS and a decrease in relative crystallinity [27].

### 3.3. Effect of HMT on Short-Range Order Structure of SPS

**Figure 3:**
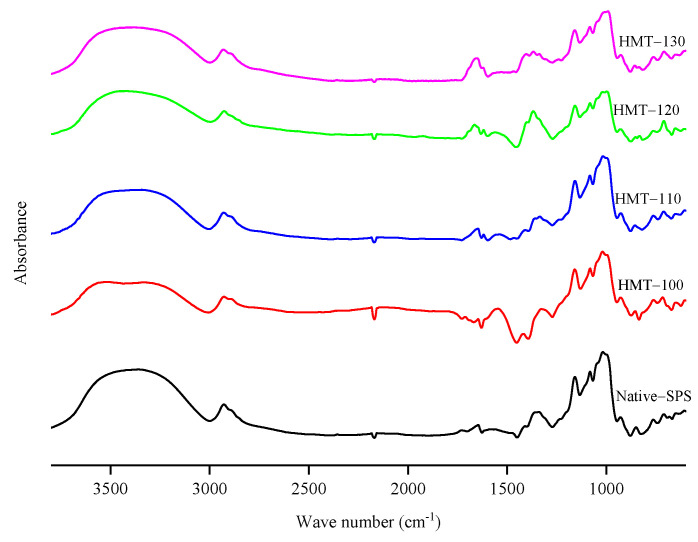
Infrared spectra of SPS before and after HMT.

**Figure 4:**
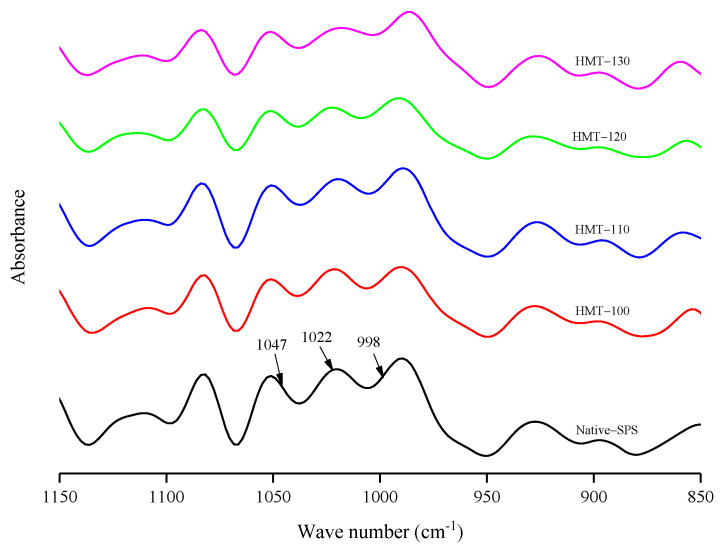
Infrared deconvolution spectra of SPS before and after HMT.

Figure 3 shows the infrared spectra of SPS before and after HMT. The starch samples had three absorption peaks at 3100–3600 cm^−1^, 3000-2800 cm^−1^ and 1640 cm^−1^, which are attributed to the bending vibrations of O-H, C-H and H-O-H, respectively [28]. The positions of the absorption peaks of the characteristic group of SPS almost did not change after HMT, indicating that no new groups or chemical bonds were formed during the reaction. Figure 4 shows the corresponding deconvolution spectra. R_1047/1022_ reflects the short-range order degree on the surface of starch particles [29,30]. As shown in Table 1, R_1047/1022_ of treated SPS was lower than that of raw starch, indicating that the short-range order of SPS particles decreased with the increase in treatment temperature. This may be because the HMT destroys the existing hydrogen bonds between or within the starch molecular chains, leading to the irregular arrangement of the double helix structure of starch molecules and the easy destruction of the spiral structure of the starch at high temperatures. Wang et al. [24] also found that with the increase in HMT temperature, the degree of short-range ordering of starch decreased more significantly.

### 3.4. Effect of HMT on the Thermal Properties of SPS

The DSC curve reflects the change in thermal stability of starch particles during heating. As shown in Table 2, HMT had a significant effect on T_o_, T_p_ and T_c_ of SPS. With the increase in temperature, T_o_, T_p_ and T_c_ of treated SPS were higher than those of raw starch. HMT degraded the amylopectin molecules inside starch particles. When the processing temperature was sufficient (100–120 °C), under the action of heat energy and water molecules, the amylopectin molecules inside the starch molecules degraded. As a result, the starch chains recombined to form an ordered structure (double helix or crystalline structure) with a higher thermal stability, which enhanced the rigidity of the starch structure, resulting in an increase in the pasting temperature of starch [31]. However, a further increase in the treatment temperature (130 °C) destroyed these ordered structures, leading to a decrease in pasting temperature.

### 3.5. In Vitro Digestion Characteristics of Starch

The different digestion rates of starch in the human body are generally divided into RDS, SDS and RS [22]. Table 3 shows the effect of HMT on the in vitro digestion characteristics of SPS. The contents of RDS, SDS and RS in raw SPS were 53.82%, 28.27% and 17.92%, respectively. After HMT, the RDS content of SPS decreased, while the SDS and RS contents increased. HMT promoted the formation of a rigid structure, hindered the decomposition rate of enzymes in starch and reduced the digestion rate [32,33,34]. When the processing temperature was 110 °C, the RS content rose to 24.77% and then stabilized as the temperature increased. During this process, the original structure of starch was destroyed and the interaction between amorphous regions and molecular chains was enhanced, resulting in an increase in RS content [10,35], which was consistent with the XRD and FTIR results. 

## 4. Conclusions

This study investigated the effect of temperature changes during HMT on the structure and in vitro digestibility of SPS. The results indicated that temperature was one of the most important factors affecting the modification of SPS, having a significant impact on the structure of SPS. After HMT, the surface of SPS granules was destroyed, resulting in fracture, depression and adhesion. Although the polarized cross of SPS was retained, the arrangement was irregular. HMT did not change the original crystallization type of sweet potato starch (A-type), but the diffraction peak at 19.8° was more pronounced. The relative crystallinity and short-range order of SPS decreased gradually, but the thermal stability increased. The in vitro digestibility of SPS changed with the increase in HMT temperature in an overall downward trend. The RS content reached its highest (24.77%) when the temperature was 110 °C at 25% moisture for 4 h. This was also the most ideal temperature for HMT of SPS. This study provides a theoretical basis for the preparation of RS using HMT and also provides a reference for the development of SPS products.

## Figures and Tables

**Table 1:** Relative crystallinity and short-range order of SPS before and after HMT.

Samples	Relative Crystallinity (%)	R_1047/1022_
Native-SPS	23.51 ± 1.69 ^a^	0.93 ± 0.01 ^a^
HMT-100	17.51 ± 0.54 ^b^	0.88 ± 0.03 ^b^
HMT-110	15.81 ± 0.12 ^c^	0.87 ± 0.01 ^b^
HMT-120	14.40 ± 0.15 ^cd^	0.84 ± 0.10 ^b^
HMT-130	12.99 ± 0.17 ^d^	0.86 ± 0.01 ^b^

**Note:** R_1047/1022_ represents the ratio of peak intensity between R_1047_ cm^−1^ and R_1022_ cm^−1^ in the infrared spectrum. Different letters indicate significant differences between data (*p* < 0.05).

**Table 2:** Thermal properties of SPS before and after HMT.

Samples	T_o_/°C	T_P_/°C	T_C_/°C
Native-SPS	69.20 ± 0.09 ^e^	74.95 ± 0.00 ^d^	84.35 ± 0.13 ^e^
HMT-100	82.02 ± 0.52 ^c^	88.37 ± 0.11 ^c^	98.14 ± 0.91 ^c^
HMT-110	83.02 ± 0.25 ^b^	89.63 ± 0.00 ^b^	99.31 ± 0.06 ^b^
HMT-120	84.94 ± 0.30 ^a^	90.16 ± 0.11 ^a^	101.53 ± 0.09 ^a^
HMT-130	70.17 ± 0.02 ^d^	75.10 ± 0.00 ^d^	86.49 ± 0.00 ^d^

Different letters indicate significant differences between data (*p* < 0.05).

**Table 3:** Effect of HMT on in vitro digestibility characteristics of SPS.

Samples	RDS (%)	SDS (%)	RS (%)
Native-SPS	53.82 ± 0.13 ^a^	28.27 ± 2.24 ^e^	17.92 ± 2.37 ^d^
HMT-100	36.88 ± 2.62 ^d^	39.59 ± 2.97 ^a^	23.54 ± 0.35 ^c^
HMT-110	37.13 ± 0.18 ^c^	38.11 ± 0.17 ^b^	24.77 ± 0.35 ^a^
HMT-120	37.99 ± 0.35 ^c^	37.86 ± 0.52 ^c^	24.15 ± 0.17 ^b^
HMT-130	39.72 ± 0.70 ^b^	35.77 ± 0.35 ^d^	24.52 ± 1.05 ^ab^

Different letters indicate significant differences between data (*p* < 0.05).

## Data Availability

The data that support the findings of this study are available on request from the corresponding author. The data are not publicly available due to privacy or ethical restrictions.

## Abbreviations

Native-SPS	native sweet potato starch
HMT-100	heat–moisture treatment-100
HMT-110	heat–moisture treatment-110
HMT-120	heat–moisture treatment-120
HMT-130	heat–moisture treatment-130
T_C_	termination temperature
SEM	scanning electron microscopy
SPS	sweet potato starch
HMT	heat–moisture treatment
RDS	rapidly digestible starch
SDS	slowly digestible starch
RS	resistant starch
T_o_	initial temperature
T_P_	peak temperature
